# Laser Spectroscopic Characterization for the Rapid Detection of Nutrients along with CN Molecular Emission Band in Plant-Biochar

**DOI:** 10.3390/molecules27155048

**Published:** 2022-08-08

**Authors:** Tahani A. Alrebdi, Amir Fayyaz, Haroon Asghar, Samira Elaissi, Lamia Abu El Maati

**Affiliations:** 1 Department of Physics, College of Science, Princess Nourah bint Abdulrahman University, P.O. Box 84428, Riyadh 11671, Saudi Arabia; 2 National Centre for Physics, Quaid-i-Azam University Campus, Islamabad 45320, Pakistan

**Keywords:** plant-biochar, CF-LIBS, CN molecular band, LTE, chemical composition, EDX, PCA

## Abstract

We report a quantitative analysis of various plant-biochar samples (S1, S2 and S3) by utilizing a laser-induced breakdown spectroscopy (LIBS) technique. For LIBS analysis, laser-induced microplasma was generated on the target surface by using a focused beam through a high-power Nd: YAG laser and optical emission spectra were recorded using a charged coupled device (CCD) array spectrometer, with wavelength ranges from 200 nm to 720 nm. The spectroscopical analysis showed the existence of various ingredients, including H, Li, Ca, Na, Al, Zn, Mg, Sr, Si, and Fe, along with a CN molecular emission band due to B^2^Σ^+^ − X^2^Σ^+^ electronic transition. By assuming conditions of the plasma is optically thin and in LTE, calibration-free laser-induced breakdown spectroscopy (CF-LIBS) was utilized for the compositional analysis of the ingredients present in the three plant-biochar samples. To lower the uncertainties, we used an average composition (%) of the three plant-biochar samples. The quantitative study of the plant-biochar samples was also achieved using the energy dispersive X-ray (EDX) technique, showing good agreement with the CF-LIBS technique. In addition, statistical analysis, such as principal component analysis (PCA), was performed for the clustering and classification of the three plant-biochar samples. The first three PCs explained an overall ~91% of the variation in LIBS spectral data, including PC1 (58.71%), PC2 (20.9%), and PC3 (11.4%). These findings suggest that LIBS is a robust tool for rapid measurement of heavy as well as light elements, such as H, Li, and nutritional metals in plant-biochar samples.

## 1. Introduction

Laser-induced breakdown spectroscopy (LIBS) can be considered an efficient, reliable, non-destructive, and versatile analytical technique, with potential applications in industry, scientific research, environmental and biological fields, and agriculture [1,2,3,4,5,6]. In the LIBS technique, the plasma emission spectra of the target sample are investigated. The plasma generated at the surface of the target, such as gas, liquid, and solid, is normally generated when the high-power laser beam is focused onto the target material [7,8,9]. The plasma emission spectrum from the laser-produced plasma gives the optical fingerprints of the sample ingredients [10,11]. LIBS is a fast, real-time, and cost-effective technique that could investigate the multiple ingredients present in the target sample both quantitatively and qualitatively [12]. However, traditional methods for compositional analysis of the samples mainly include X-ray fluorescence spectroscopy (XRF) [13], inductively coupled plasma mass spectrometer (ICP-MS) [14], inductively coupled plasma-atomic emission spectroscopy (ICP-AES) [15], and atomic-absorption spectroscopy (AAS) [16].

The calibration-free LIBS (CF-LIBS) technique has already been extensively utilized for the compositional studies of solids, liquids, and gaseous samples without using calibration standards and avoiding matrix effects, as described by Ciucci et al. [17]. The standard-less CF-LIBS procedure has lots of potential uses compared to the calibration curve LIBS (CC-LIBS) for quantitative study in scientific research and industry, and for the analysis of plant biochar, coal, soil, cement, alloys, geological and biological samples [17,18,19]. For the use of the CF-LIBS technique, a few assumptions must be assured, such as stoichiometric laser-ablation, plasma should be homogeneous (spatial and temporal), optically thin, and follow the local thermodynamical equilibrium (LTE) state [17]. Furthermore, for compositional analysis of samples relative to traditional techniques (XRF, ICP-MS, ICP-AES, AAS), energy-dispersive X-ray (EDX) is an investigative tool that is commonly used for the elemental or chemical characterization of substances. In EDX, X-rays are produced by using a high-power electron-beam that bombards the surface of the sample [19]. An electron transmits its energy to the atomic electron of the target material. The electron gets excited by absorbing the energy and is knocked out from the shell by leaving behind a vacancy. An electron jumps from the higher shell to fill the available vacant state in the lower-energy shell and a photon, with energy corresponding to the difference (ΔE) in this transition energy, is released in the form of X-rays. By applying this technique, the characteristic energies of each element, along with the relative intensities of the corresponding X-ray signals, are used for the compositional studies of the ingredients that are present in a target. Moreover, to overcome the complications of the matrix effect for the quantitative analysis and classification of the samples, various algorithms, including artificial neural network and principal-component-analysis (PCA), were also realized. PCA is an unsupervised machine learning and the statistical algorithm technique used to lower the high dimensional data to a set of low dimensional correlated variables. These low-dimensional correlated variables are known as principal components (PCs) [20]. In the present study, PCs were constructed using the maximum covariance in the spectral data. This is achieved through low-ranked, PCs while high-ranked PCs were ignored. These low-ranked principal components are capable of maintaining the most significant characteristics of the spectral data. Consequently, the acquired spectral data are normalized (by dividing the spectral data by the standard deviation). After normalization, PCA is utilized in the spectral LIBS data-set to reduce the dimensions and transform the entire spectral data (intensity against wavelength) into several principal components (PCs).

Biochar is generally a rich organic substance that contains carbon (C) manufactured via plants and animal waste, which is pyrolyzed under an oxygen (O_2_)-controlled environment [21,22]. The various physical characteristics of biochar, including surface area, pore capacity, and ore allocation, are highly affected by the numerous pyrolysis conditions and biomass waste [23]. Biochar is a blackened substance, similar to charcoal material, due to a relatively high concentration of carbon (C) material, minimal nutrient substance, and high surface area. However, it is different from charcoal because it is utilized to control soil fertility and crop production [24]. The fruit, vegetable, and animal waste materials are naturally bio-degradable; however, they cause environmental contaminants and impurities. Pyrolysis and composting methods are commonly utilized tools for reprocessing these plants and animals’ waste into useful organic goods to enhance soil strength and crop production [25]. Chan et al. [26] found that the use of biochar reduced the yielding strength of soil cores, showing that biochar can decrease the risk of soil compaction and increase the pore volume. Verheijen et al. [27] reported that the biochar impeded the toxicity in the soil. Verheijen et al. further studied the effect of various heavy metals in the soil owing to improved soil concentration power. Liu et al. [28] studied the IR bands among biochars and Fourier transform infrared (FTIR) technique was further employed to characterize the biochar structure. In addition, it was also reported that PCA of FTIR spectra indicated similarities and dissimilarities of biochar samples prepared at different pyrolysis temperatures. Most recently, Duan et al. [20] employed a calibrated LIBS technique on agriculture biochar for the quantitative studies of heavy elements, including Cr, and Pb, along with major nutritional metals, including Cu, Zn, Ca, Na, K, and Mg.

In the present study, various plant waste materials were converted to plant-biochar samples, and the compositional studies were acquired utilizing the CF-LIBS technique. The carbon (C), calcium (Ca), and zinc (Zn) in the three plant-biochar samples were successfully identified as major elements using LIBS. Likewise, the quantitative results obtained using CF-LIBS were compared with that determined from EDX, showing good compatibility between these two analytical techniques. In addition, PCA was carried out for clustering, and the classification of the three plant-biochar samples based on the optical emission lines of Zn (I), Ca (II) and C (I) at 481.05 nm, 393.37 nm, and 247.86 nm for S1, S2, and S3 samples, respectively. PCs described an overall ~91% of the variation, including PC1 (58.71%), PC2 (20.9%), and PC3 (11.4%). This study suggests that LIBS, in combination with EDX and PCA, is a reliable tool to monitor the presence of nutrients that exist in the soil for rich crop production, soil amendment, and better soil health and has potential applications in agriculture.

## 2. Experimental Detail

**Figure 1:**
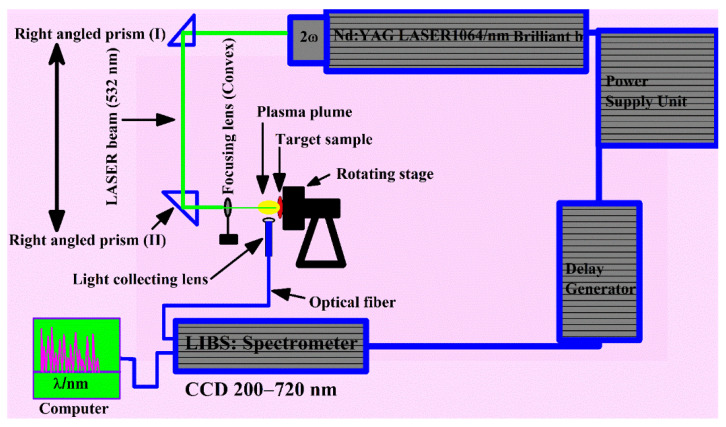
Schematics of LIBS setup utilized to investigate the laser-produced plasma emission of the plant biochar samples.

The LIBS experimental arrangements employed to investigate the compositional analysis of the various samples have been explained elsewhere [29,30,31]. A schematic of the LIBS arrangement used to study the plant-biochar samples is presented in Figure 1. In brief, the LIBS experimental setup contains an Nd: YAG pulsed laser system, which produces light at the wavelength 532 nm, operates at 10 Hz repetition frequency, 5 ns pulse duration, and is capable of delivering the pulse energy of ~200 mJ. The laser beam, with 90 mJ pulse energy, and 0.018 GW power, was focused onto the surface of the target material, placed at atmospheric pressure in the air through a convex (quartz) lens with a 10 cm focal length. The plant-biochar material was kept on a revolving circular stand to enable a clean target surface for each fresh laser pulse. The optical emission spectra were recorded using an optical fiber attached to a charged coupled devices (CCDs) array spectrometer, with 200–720 nm wavelength. An average of ten laser shots were used to clean the target surface. The spectra from plasma optical emission were accomplished at 20 average laser shots on the target surface of the sample. The averaged optical spectra from plasma emission were then utilized to lower the statistical errors and sample inhomogeneity.

EDX attached to a scanning electron microscope (SEM) is a robust technique for exploring biological, geological, and mineralogical samples with a complex distribution matrix, and it can also be appropriate to detect toxic elements in medicinal and plant-biochar samples with low concentrations. For the EDX analysis, an Oxford EDX Instrument (X-MAXN-20), coupled with a scanning-electron-microscope (SEM), with a depth profile of 1–2 µm that operated at ~20 keV beam energy, was utilized to study the chemical composition of the plant-biochar target samples. The X-rays emitted from the surface of the target sample were identified through a detector (30 mm^2^ Si (Li)) [19].

## 3. Material

The plant waste material was collected from northern areas of Pakistan and converted to plant-biochar material using slow pyrolysis under an oxygen (O_2_) controlled environment. The pyrolysis technique involves the chemical process to heat organic material, such as biomass (waste material), under a low oxygen (O_2_) environment at about 400 °C temperature. The thermal decomposition of the biomass yields a solid char, typically known as biochar. In the present work, various plant waste materials were used for the preparation of biochar samples. The biomass material with different growth conditions was collected from northern areas of Pakistan with the coordination of a local waste management company (LWMC). Three samples (S1, S2 and S3) of the plant-biochar were made using slow pyrolysis of raw material and a typical pyrolysis chamber. Initially, the selected plant raw material was dehydrated in a furnace at 100 °C for 20 h. The pyrolysis method was performed in a hermetic seal flask, comprising two metal tubs, at 400 °C. The distance between the tubs was stirred with naturally occurring gas. The biochar was produced in 4 h. and was left to cool off for 3 h, yielding 80% of the raw material biomass to biochar. Although the biochar samples were prepared under the same experimental conditions, they have different elemental concentrations, due to the different growth conditions of the plant biomass material. Having established the different elemental concentrations, as well as signal intensities of the samples using LIBS, these samples were classified as S1, S2, and S3 using PCA analysis.

## 4. Results and Discussions

### 4.1. LIBS Emission Studies

**Figure 2:**
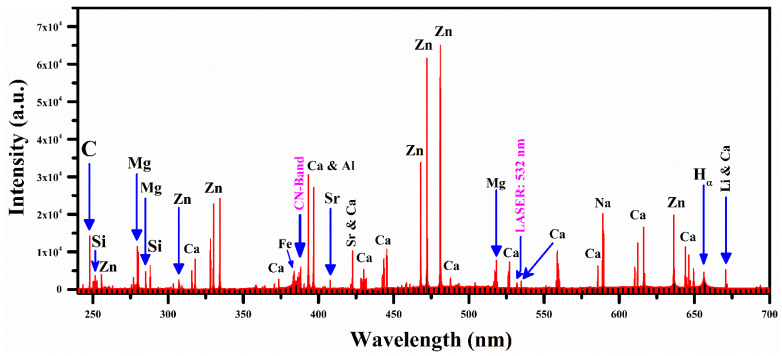
The plasma emission spectrum of the plant-biochar sample (S1) in wavelength ranges from 240 to 700 nm.

The emission spectra of the three plant-biochar samples were registered at optimized experimental conditions, including 90 mJ laser pulse energy, 0.018 GW power, 10 cm focal length of the laser beam focusing convex lens, 2 mm distance from the plasma plume to the light collecting lens and the target surface was held perpendicular to the laser light beam. The laser pulse was collimated onto the pallets of the target samples to produce the plasma at a ~90 mJ laser energy. The plasma optical emission spectrum from the laser-generated plasma was captured using a CCDs spectrometer. The spectrum was further investigated by labeling the spectral lines identified using the National Institute of Standards and Technology (NIST) database [32] and LIBS info [33]. The optical emission spectral lines of various elements with different line intensities were detected in the emission spectra of the plant-biochar samples, whereas rich emission lines of zinc (Zn) around 325 nm and 475 nm were identified in all three samples. Therefore, only one spectrum in the wavelength range from 240–700 nm is presented in Figure 2, which shows the strong as well as weak emission lines, including elements lithium (Li), hydrogen (H), sodium (Na), calcium (Ca), aluminum (Al), iron (Fe), carbon (C), strontium (Sr), zinc (Zn), and silicon (Si), along with a multiplet cluster of singly ionized magnesium (Mg II), due to the 3p ^2^P_1/2_,_3/2_ → 3s ^2^S_1/2_ and 3d ^2^D_3/2,5/2_ → 3p ^2^P_1/2,3/2_ transitions. The detected emission spectral lines of various elements present in the plant biochar sample (S1) are enlisted in Table 1.

Furthermore, the strong resonance emission lines of ionic strontium (Sr II) confirm the presence of Sr metal in all the plant-biochar samples at 407.77 nm and 421.55 nm due to the 4p^6^5p ^2^P_3/2_ → 4p^6^5s ^2^S_1/2_ and 4p^6^5p ^2^P_1/2_ → 4p^6^5s ^2^S_1/2_ spectral transitions, respectively. The plants may have absorbed Sr from the ground in which it is growing in organic environments. Strontium (Sr) metal is not an indispensable ingredient to plants or other medicinal plants. For this reason, it was observed that strontium minimized the concentration of phytoestrogen compounds and decreased the antioxidant strength [34]. Antioxidant strength describes the potency of a plant to scavenge the reactive oxygen groups, such as peroxides, singlet oxygen, superoxide, alpha-oxygen, and hydroxyl radical that enhance the therapeutic capacity of medicinal plants. Kabata-Pendias and Mukherjee reported that the concentration of strontium in plants varies from below 1 to 10^4^ mg kg^−1^ [35].

**Figure 3:**
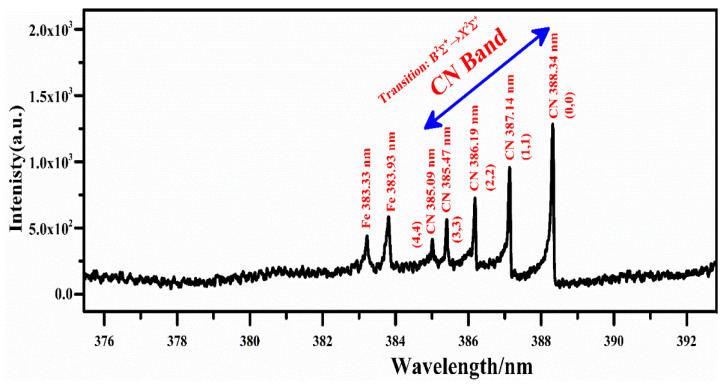
CN molecular emission spectrum due to the vibrational transitions; (0-0), (1-1), (2-2), (3-3) and (4-4), with a wavelength range from 375 nm to 393 nm.

In addition, the molecular emission structure of the CN violet band is observed in the emission spectra of biochar ranging from wavelength 384 to 390 nm, as shown in Figure 3. This figure shows that the molecular emission of a violet system of molecule diatomic CN relates to radiative transitions from B^2^Σ^+^ to X^2^Σ^+^ of the electronic state ∆ν = 0 at 3.32 eV and 0 eV, respectively. The spectral emission lines of this CN molecular band were detected at 388.34, 387.14, 386.19, 385.47 and 385.09 nm associated with the vibrational transitions (0,0), (1,1), (2,2), (3,3) and (4,4), respectively. The identification of the CN band is essential for estimating the concentration of carbon (C) and nitrogen (N). In this spectral range, the emission line of Zn II is also detected with a good signal to noise ratio (SNR) at 255.79 nm, due to 5s ^2^S_1/2_ → 4p ^2^P_3/2_ transition, and Zn I at 307.59, 328.23, 330.28, 334.53, 468.01, 472.22, 481.05, 280.12, and 636.23 nm, due to 4s4p ^3^P_1_ → 4s^2 1^S_0_, 4s4d ^3^D_1_ → 4s4p ^3^P_0_, 4d ^3^D_1_ → 4p ^3^P_1_, 4d ^3^D_3_ → 4p ^3^P_2_, 5s ^3^S_1_ → 4p ^3^P_0_, 5s ^3^S_1_ → 4p ^3^P_1_, 5s ^3^S_1_ → 4p ^3^P_2_, 5d ^3^D_1_ → 4p ^3^P_2_, 4d ^1^D_2_ → 4p ^1^P_1_ transitions, respectively. However, it is worth mentioning that various structures of Ca emission lines at 315.89, 317.93, 393.37, 396.85, 442.54, 443.50, 445.48, 527.03, 558.20, 558.88, 559.01, 559.45, 559.85, 612.22, 616.13, and 616.22 nm with sufficiently high spectral line intensities are identified, showing good concentrations of Ca in the plant-biochar samples.

### 4.2. Plasma Excitation Temperature (T_e_)

The plasma excitation temperature was approximated from the relative plasma emission line intensities of the neutral calcium (Ca) line profiles for samples (S1, S2 and S3) using the Boltzmann equation. The neutral zinc (Zn) emission line profiles for sample S1 were also used to calculate the plasma excitation temperature using the same Boltzmann plot method. The spectral emission lines utilized to structure the Boltzmann plots are shown in Table 2. The spectroscopical atomic parameters, such as optical wavelength (nm), upper-level energy (cm^−1^), transition probability (s^−1^), and statistical weight (g_k_), were used from the NIST database [32]. Generally, errors in the plasma excitation temperature using the Boltzmann plot method occurs due to the uncertainties in the calculated line intensities and transition probabilities. Therefore, in the present study, the calculated plasma excitation temperature consists of about ~5% relative error. Self-absorption in LIBS analysis lowers line broadening and the line intensity of an emission line, due to the light radiated by an atom in a laser-produced plasma being absorbed by that atom in another section of the plasma. The self-absorption effect normally generates inhomogeneous plasma, which produces several strong fluctuating slumps on the emission spectrum. This effect leads to uncertainty in measuring the compositions using CF-LIBS. In this study, we have corrected the selected emission line intensities for CF-LIBS to minimize the self-absorption effect, which is less than 5% [36,37].

By assuming that the population of plasma obeys the Boltzmann distribution, we used the following Boltzmann equation to construct the Boltzmann plots [38,39]:

(1) $$\ln\left(\frac{I_{ij}\lambda_{ij}}{hcA_{ij}g_{i}}\right)=-\frac{E_{i}}{kT_{e}}+ln\left(\frac{N_{e}}{P\left(T_{e}\right)}\right)$$

Here, *I_ij_* is the spectral intensity of the observed line *j* → *i*, λ is the optical wavelength of the transition, *h* denotes the Planks constant, *A_ij_* represents the reported absolute transition-probability, $g_{i}\;$is the upper-level statistical-weight, *c* indicates the velocity of light, $E_{i}$ is the upper-level energy, k represents the Boltzmann constant parameter, *T_e_* is the plasma excitation temperature, $N_{e}$ is the total population density, and P(T) is the temperature-dependent partition function.

**Figure 4:**
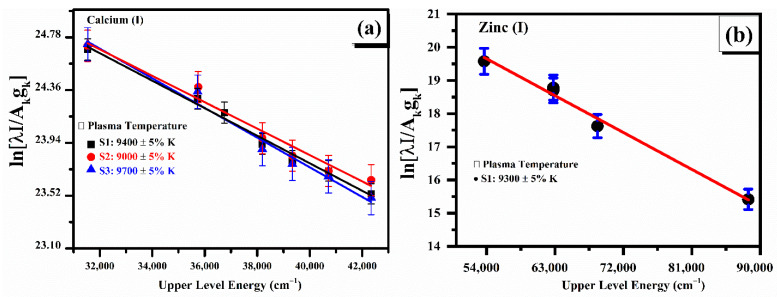
(**a**) Typical Boltzmann plots for the three plant-biochar samples (S1, S2 and S3) using several neutral emission lines of Ca (I); (**b**) shows the Boltzmann plots for sample (S1) using zinc (I) emission lines.

Figure 4a reveals typical Boltzmann plots of the three plant-biochar samples (S1, S2 and S3) using the neutral Ca (I) lines, which are corrected from the self-absorption effects. The slopes (1/k_B_T) relate to the linear data fitting in the Boltzmann plots that provide the plasma excitation temperature. The plasma electron temperature determined from Ca (I) spectral lines was (9400 ± 5%) K for S1, (9000 ± 5%) K for S2, and (9700 ± 5%) K for S3 samples of the plant-biochar. The average value of the plasma temperature estimated for these three samples was (9366 ± 5%) K. Similarly, the calculated plasma excitation temperature from the Zn (I) emission lines is presented in Figure 4b as (9300 ± 5%). The error bars show the estimated error, which is attributed to the errors present in the integrated line intensities, and absolute transition probabilities. The calculated uncertainty in the measurement of electron temperature is ∼5%. Interestingly, the estimated average value of the temperature from Ca lines and for Zn (I) emission lines are in good agreement. Therefore, the average plasma excitation temperature (9366 ± 5% K) was employed to measure the chemical compositions of various plant-biochar samples.

### 4.3. Plasma Electron Density (N_e_)

The plasma electron number density is approximated using an isolated Stark-broadened Ca I spectral line at wavelength 610.27 nm and the Stark-broadened emission line profile of the hydrogen (H_α_) at wavelength 656.28 nm. The full width at half maxima, *Δλ_1/2_* (*FWHM*), of Ca and H_α_ spectral lines was obtained by the deconvolution process using the Voigt fitting line profile with instrumental width (0.05 ± 0.01 nm), the Doppler width (0.005 nm) and the Stark width (ω_s_). The relation for the Stark width in terms of plasma electron density and *FWHM* is given as follows [40,41]:

(2) $$N_{e}\left({\mathrm{cm}}^{-3}\right)=\frac{\Delta \lambda_{1/2}\left(FWHM\right)}{2\omega_{s}\left(\lambda ,\;T_{e}\right)}\times 10^{16}$$

**Figure 5:**
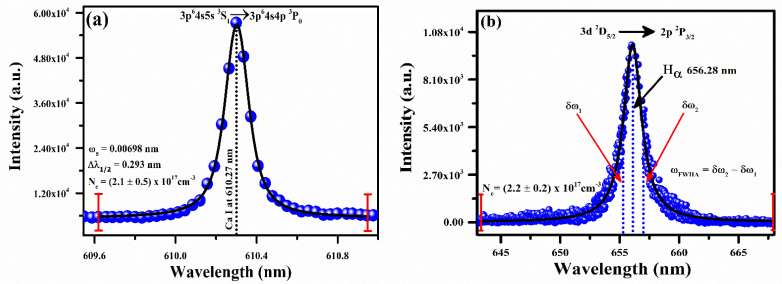
(**a**) Stark-broadened line profile of Ca I spectral line at wavelength 610.27 nm; (**b**) H_α_ spectral line of hydrogen at wavelength 656.28 nm, showing full width at half area (*FWHA*).

Here, *N_e_* represents the plasma electron number density. The Stark broadening parameter (*ω_s_*) is a partial function of optical wavelength (*λ)* and excitation temperature (*T_e_*). Figure 5a demonstrates the Stark-broadened spectral line profile of the Ca I spectral line at 610.27 nm. The emission spectrum was obtained under optimized conditions, such as ~90 mJ laser pulse energy and a 2 μs delay time among laser pulse and spectral data acquisition. The data points (blue color) signify the experimental measurements and the solid black line shows the Voigt fitting line profile, which gives the full width at half maxima (*FWHM*) as (0.321 ± 0.5) nm. Taking into consideration the 0.00698 nm reported Stark-broadened parameter, the plasma electron density *N_e_* is measured to be $\left(2.1\pm 0.5\right)\times 10^{17}$ cm^−3^.

The full width at half area (*FWHA*) is calculated using a H_α_ of a hydrogen emission spectral line at wavelength 656.28 nm, as demonstrated in Figure 5b. The observed line profile (red color) data and two dotted vertical lines correspond to *FWHA* yielding as (0.94 ± 0.05) nm. The simplest formula for the calculation of plasma electron density using H_α_ line is given as follows [42,43]:

(3) $$\omega_{FWHA}=5.49Å\times {\left(\frac{N_{e}}{10^{17}{\mathrm{cm}}^{-3}}\right)}^{0.67965}$$

Here, *N_e_* represents the plasma electron density, and ω*_FWHA_* is the full width at half area. The parameter ω*_FWHA_* is calculated through the relation, $\omega_{FWHA}=\delta \omega_{2}-\delta \omega_{1}$, as demonstrated in Figure 5b. The calculated plasma electron density using Equation (3) is $\left(2.2\pm 0.2\right)\times 10^{17}$ cm^−3^. The measured error in the electron number density is ∼5%, which mostly appears due to the errors in the deconvolution of the line width to the instrumental width, electron impact parameter, and *FWHM* from the fitting profile. Excellent compatibility is observed among the calculated densities using the Stark Broadening spectral line profile of Ca I at wavelength 610.27 nm and the hydrogen H_α_ emission line at wavelength 656.28 nm. We used an average value of number densities as $\left(2.15\pm 0.7\right)\times 10^{17}$ cm^−3^ to estimate the compositions of the plant-biochar samples using CF-LIBS.

### 4.4. Optically Thin and Local Thermodynamical Equilibrium (LTE) Conditions

For the CF-LIBS elemental analysis, laser emitted plasma should be optically thin, stoichiometric, self-absorption free, and follow the local thermodynamical equilibrium (LTE) condition. To confirm the conditions of being self-absorption free and optically thin, we used the intensity ratio technique [36,44]. For instance, the corrected line intensities of Ca, Mg, and Fe, containing approximately the same upper-level energies, were used to reduce the electron temperature dependency. The experimentally observed intensity ratios of the spectral lines and theoretically measured values from the atomic parameters (NIST database 2022 [32]) are quite compatible within about 10% uncertainty.

The excitation temperature and the ionic temperature have to be equal to the electronic temperature for the plasma to be in LTE. McWhirter suggested the principle of a minimum limit to the electron number density to verify the plasma proximity to the LTE. The minimum limit for the electron number density is determined using the following relation [39,45,46]:

(4) $$N_{e}\left({\mathrm{cm}}^{-3}\right)\geq 1.6\times 10^{12}\sqrt{(T_{\left(K\right)}}{\left(\Delta E\;\left(eV\right)\right)}^{3}$$

where ΔE(eV) is the highest transition energy among the upper as well as lower levels, and $T_{\left(k\right)}$ represents the plasma excitation temperature. In this work, the calculated plasma electron density using this relationship is on the order of ~$10^{14}{\mathrm{cm}}^{-3}$. This value of the plasma electron density is lower at three orders of magnitude than that determined from relations (2) and (3). Therefore, it can be concluded that plasma satisfies the LTE condition.

The validity of the LTE condition has also been confirmed by estimating the diffusion length using the Cristoforetti et al. [47] criterion for an inhomogeneous plasma to be in LTE. According to this condition, the characteristic variational length “*d*” is too high compared to the diffusion length (10λ<d). The diffusion length was estimated by utilizing the following relations [47,48].

(5) $$\lambda \approx \sqrt{D_{diff}\times \tau_{rel}}=1.4\times 10^{12}\;\left[\frac{{\left(k_{B}T\right)}^{\frac{3}{4}}}{N_{e}}\right]\cdot \;{\left(\frac{\Delta E}{M_{A}\;f_{12}\;\left(\bar{G}\right)}\right)}^{\frac{1}{2}}\cdot \exp\left[\frac{\Delta E}{2k_{B}T}\right]$$

(6) d≈T(x)(dT(x)dx)−1

where *k_B_T* and Δ*E* are determined by electron volt (eV), *N_e_* denotes the plasma density measured in (cm^−3^), *M_A_* represents the atomic mass of a specie, *f_12_* indicates the atomic oscillator strength taken from the NIST database [32], $\bar{G}$ is the gaunt factor carefully chosen by Cristoforetti et al. [48] and *d* is the plasma diameter (typically a few mm), also known as characteristic variation length [48]. In the present work, we calculated the diffusion length by utilizing the emission line of neutral calcium (Ca I). The calculated diffusion length, $\lambda ≅1.66\times 10^{-3}\;\mathrm{mm}$, is much lower than the characteristic variational length of the plasma (10λ<d), which ensures that the plasma is much closer to the LTE. Once the plasma is found to be optically thin and in LTE, the plasma parameters can be utilized to approximately calculate the elemental composition using the CF-LIBS technique.

## 5. Principal Component Analysis (PCA)

PCA is an inherent un-supervised machine learning tool usually applied to high-ranking dimensional data. PCA test remodels the dataset from high-ranking dimensions to significant low-dimension correlated variables known as principal components (PCs). PCA can be applied for sample classification and high dimension reduction using a scoring matrix [20,49,50].

**Figure 6:**
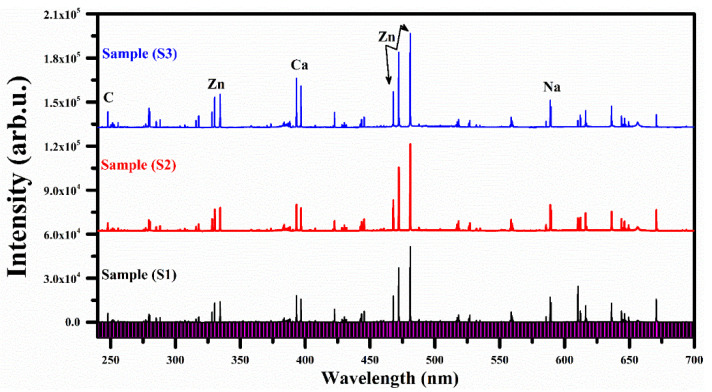
Average LIBS spectra of plant-biochar samples (S1, S2 and S3).

The averaged spectra of the three plant-biochar samples (S1, S2 and S3) in the spectral region ranging from wavelength 240 to 700 nm are presented in Figure 6. Various spectral similarities were detected from the three LIBS spectra of the plant-biochar samples, showing the same elemental components. After pre-processing of the LIBS data using background correction, 13 spectra were selected as an average of 100 spectra for each plant-biochar sample. The selected spectral data are normalized by dividing the data set by the standard deviation (SD) of the individual spectral data.

**Figure 7:**
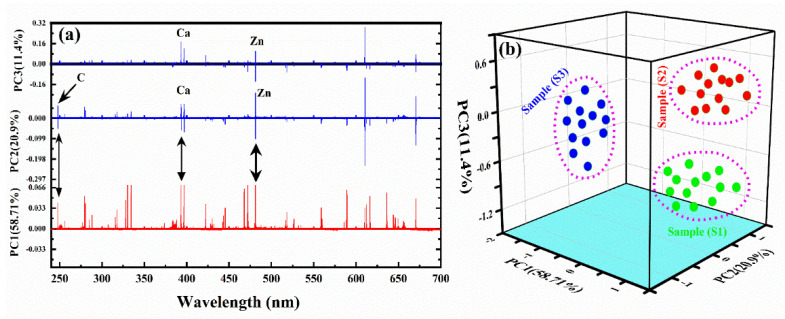
(**a**) The loading plots of the first 3 PCs, (**b**) 3D plot of the intensity of Zn (I) at wavelength 481.05 nm, Ca (II) at wavelength 393.37 nm, and C (I) at wavelength 247.86 nm for S1, S2, and S3, respectively.

In Figure 7a, we present the loading plots of the first three PCs using LIBS spectral data. It can be observed that the spectral emission lines of the main elements, such as C, Ca, and Zn, are in good alignment, presenting relatively large loading coefficients. In addition, those spectral lines that have a high signal-to-noise ratio (SNR) were carefully chosen to draw the 3D cluster plot. In the present work, we selected the line intensity of Zn (I) at a wavelength of 481.05 nm, Ca (II) at a wavelength of 393.37 nm, and C (I) at a wavelength of 247.86 nm for S1, S2, and S3, respectively. A 3D cluster plot was constructed with limited input variables for the classification of the three plant-biochar samples, as shown in Figure 7b. In Figure 7b, the first three PCs described an overall 91% variation for the full LIBS spectral data, including 58.71%, 20.9%, and 11.4% for PC1, PC2, and PC3, respectively. The three distinct clusters, S1, S2, and S3, show three different well-separated plant-biochar samples based on C, Zn, and Ca emission lines. Therefore, the spectral data of the three elements C, Ca, and Zn in the plant-biochar samples indicate that these emission lines can be useful for the classification of the samples.

## 6. Chemical Composition by CF-LIBS and Comparison with EDX

We employed the calibration-free (CF-LIBS) technique in order to estimate the chemical composition of the three plant-biochar samples, which is discussed in detail elsewhere [51,52,53]. The atomic chemical composition of the neutral atoms is measured by utilizing a well-known Boltzmann relation [54,55].

(7) $$FW^{\alpha ,\gamma }=I_{ki}\frac{U^{\alpha ,\gamma }\left(T\right)}{A_{ki}g_{k}}\exp\left[\frac{E_{k}}{k_{B}T}\right]$$

Here, $U^{\gamma }\left(T\right)$ is the partition function that is defined as $U^{\gamma }\left(T\right)=\underset{i}{\sum }g_{i}e^{-\frac{E_{i}}{k_{B}T}}$, $W^{\gamma }$ is the concentration of the neutral atom, factor *F* represents the ablated mass volume and constant-efficiency parameter of the spectral system. Ablation mass parameter *F* can be achieved by normalizing the concentrations of elements present in the sample [26,50], $I_{ki}$ is the integrated transition line strength, $g_{k}$ represents the statistical weight, $A_{ki}(s^{-1}$) denotes the transition probability from (k→i), *E_k_* (eV) is the upper-level energy, *T* is the excitation temperature in (eV), and *k_B_* denotes the Boltzmann constant in eV/K. All the atomic factors utilized for the investigation were taken from the NIST database [32]. The concentrations ($W^{\gamma }$) of the neutral atoms in the sample are calculated using Equation (7). To minimize the relative error, we used the average value of electron number density and plasma excitation temperature for the CF-LIBS analysis. To calculate the chemical composition of the ionic species present in the samples, the Saha–Boltzmann equation was applied and related to the concentrations in the two consecutive charge states *γ* and *γ* + 1 of a particular element *α* [17,41,52,56].

(8) $$N_{e}\frac{W^{\alpha ,\;\gamma +1}}{W^{\alpha ,\gamma }}=6.04\times 10^{21}\;\sqrt[{3}]{T_{eV}}\;\frac{U_{\alpha ,\gamma +1}}{U_{\alpha ,\gamma }}\;\exp\left[-\frac{E_{\alpha ,\gamma }}{k_{B}T}\right]$$

Here, $W^{\alpha ,\;\gamma +1}$ is the concentration of the *γ* + 1 charge state, $E_{\alpha ,\gamma }$ is the ionization energy of the element *α* in (eV), $N_{e}\;\left({\mathrm{cm}}^{-3}\right)$ represents the plasma electron density, $U_{\alpha ,\gamma +1}$ and $U_{\alpha ,\gamma }$ are the partition functions of the upper-charge state (*γ* + 1) and lower-charge state (*γ*), respectively. The composition of any element in a sample is the total of the neutral ($W^{\alpha ,\gamma }$) and ionized ($W^{\alpha ,\;\gamma +1}$) contributions [57].

(9) $$W_{total}^{\alpha ,\gamma }=W^{\alpha ,\gamma }+W^{\alpha ,\gamma +1}$$

The experimental parameter *F* can be approximated by normalizing the total concentration of all the ingredients to achieve unity, for example, $\sum W_{total}^{\alpha ,\gamma }=1$. We employed the following equation to measure the percentage elemental composition of all the elements present in the plant-biochar samples.

(10) $$W^{\alpha }\left(\%\right)=\frac{W^{\alpha }}{\sum W_{total}^{\alpha ,\gamma }}\times 100$$

where $W_{total}^{\alpha ,\gamma }$ represents the relative chemical weight of each component and $\sum W_{total}^{\alpha ,\gamma }$ is the sum of concentrations of all components present in the plant-biochar samples. Relative standard deviation (RSD) (coefficient of variation) is the calculation of accuracy in data analysis. The relative standard deviation (RSD) of statistics closely belongs to the standard deviation (SD). Standard deviation provides the variance in individual spectral data from the mean of the data points. We determined the average composition of the three plant-biochar samples (S1, S2 and S3) by taking the average spectra of each sample under identical conditions and optimized LIBS parameters. Therefore, to reduce the relative error in composition (%), we calculated the relative standard deviation error (*RSDE*) in the composition (%) of each chemical component in the sample [58,59].

(11) $$\sigma =\sqrt{\frac{\Sigma {\left(\left(y_{i}-\Omega \right)\right)}^{2}}{n}}$$

(12) $$RSDE\;\left(\%\right)=\frac{\sigma }{\Omega }\times 100$$

Here, σ is the standard deviation, $y_{i}$ is the value from the data, Ω is the average value of the data and *n* indicates the total number of the values.

There are 17 major and minor essential constituents for plant nutrition and growth, such as hydrogen (H), carbon (C), molybdenum (Mo), oxygen (O), copper (Cu), phosphorus (P), nitrogen (N), potassium (K), magnesium (Mg), sulfur (S), chlorine (Cl), calcium (Ca), boron (B), iron (Fe), manganese (Mn), zinc (Zn) and nickel (Ni) [60]. We found some essential and non-essential (toxic and non-toxic) ingredients, including H, Li, Mg, Na, Al, Si, Ca, Sr, C, Zn, and Fe, in the plant-biochar samples using the LIBS technique.

**Figure 8:**
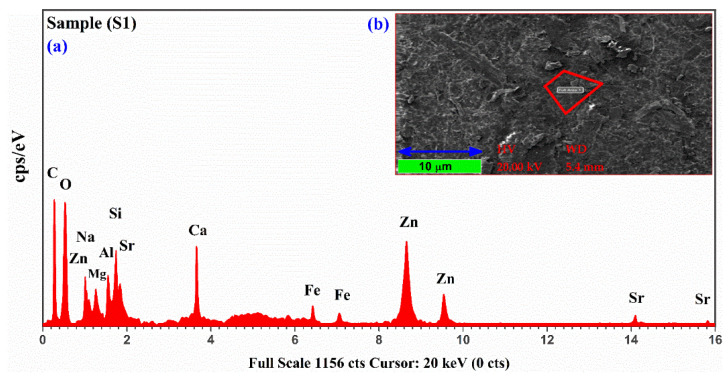
(**a**) Energy dispersive X-ray spectrum of the plant-biochar sample (S1); (**b**) shows the microphotograph of the surface of the sample (S1) with a specific scanned area (red closed path).

Energy dispersive X-ray (EDX) is a standard analytical method for the identification of trace ingredients in geological, mineralogical, and environmental samples. EDX can detect various elements, such as C, O, Si, Pb, Na, Mg, Al, Cu, Cl, Ni, K, Ca, Ti, Fe, P, Cr, Mn, Cd, Zn, As, Se, Ba, Sr, and Co, in soil and plants. The microphotograph and corresponding EDX spectrum were taken for all three samples (S1, S2 and S3). However, we present only one typical microphotograph and EDX spectrum of the sample (S1) in this study. In Figure 8a, we present the EDX spectrum of the plant-biochar sample (S1). Figure 8b shows the microphotograph of EDX, which was recorded using an accelerating voltage of 20 keV under a high vacuum (~10^−7^ mbar), along with a resolution up to 10 μm. The maximum magnification is around $\sim 3\times 10^{5}$ times higher than what is possible in this equipment with the high energy of ~130 eV. The EDX analysis shows the presence of C (21.44%), Na (2.58%), Mg (4.97%), Al (2.43%), Si (2.87%), Ca (27.88%), Fe (3.27%), Zn (31.89%), and Sr (2.67%) as the major (primary), minor (secondary), and trace (micro) nutrients of the plant-biochar sample (S1). Interestingly, elements detected by the LIBS technique are also present in the EDX spectrum except for Li, the lightest element with a low X-ray yield, as compared to the other detected components. After bombarding an accelerated (1–10 kV) beam of electrons onto the Li atoms, Li releases auger electrons instead of photons with discrete kinetic energies. Therefore, EDX is unable to detect the light elements; H, Li, Be, and B have a low X-ray yield.

**Figure 9:**
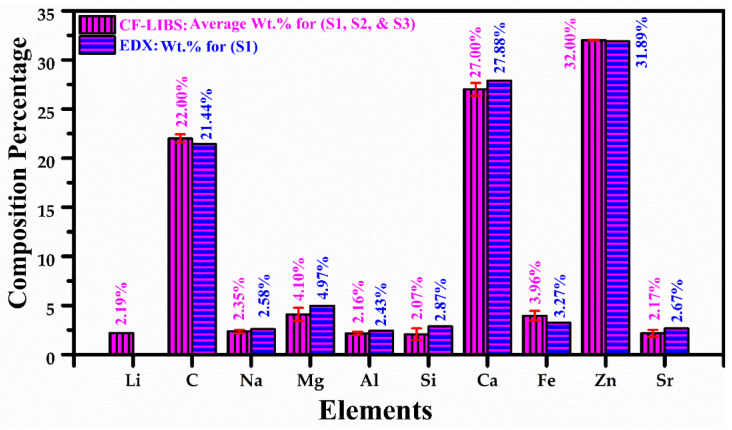
Bar graph showing average elemental composition percentage of the plant-biochar samples (S1, S2, and S3) using CF-LIBS technique and comparison with EDX.

The estimated average elemental composition (%) by the CF-LIBS method shows that C (~22%), Zn (~32%), and Ca (~27%) are the major ingredients of the plant-biochar samples. Similarly, other detected minor ingredients of the three samples are Li (2.19%), Na (2.35%), Al (2.16%), Si (2.07%), Mg (4.10%), Fe (3.96%), and Sr (2.17%). In Figure 9, we present the average relative chemical composition (%) of the detected essential and non-essential elements of the three samples determined using the CF-LIBS technique, along with the chemical composition (%) calculated by utilizing the EDX analytical method for the sample (S1). The relative standard deviation error (RSDE) of the average chemical composition (%) was determined to enhance the efficiency of the CF-LIBS technique. The error bars (red color) within ~10% RSDE in the calculated elemental composition show that the CF-LIBS method is comparable with that measured by the EDX standard analytical technique. The CF-LIBS error usually appears due to the uncertainties in the Stark width parameter, self-absorption, transition probability, and optical thickness.

## 7. Conclusions

Compositional studies of the three plant-biochar samples were carried out using CF-LIBS and EDX techniques. The time-integrated plasma emission spectra of the plant-biochar samples using LIBS confirmed the existence of major ingredients, such as carbon (C), calcium (Ca), and zinc (Zn), followed by hydrogen (H), silicon (Si), sodium (Na), magnesium (Mg), aluminum (Al), lithium (Li), iron (Fe), and strontium (Sr). For the cross-validation, EDX analysis of the plant biochar samples further confirms the presence of 9 ingredient elements, including C, Na, Al, Si, Zn, Ca, Mg, Sr, and Fe. The elements detected by the LIBS technique were also identified in the EDX analysis, except for H, and Li because EDX is unable to detect the light ingredient elements, including H, Li, Be, and B has a low X-ray yield. The quantitative analysis of the three plant-biochar target samples was achieved utilizing calibration-free laser-induced breakdown spectroscopy (CF-LIBS). The average composition (%) results, including C (22%), Ca (27%), Li (2.19%), Na (2.35%), Al (2.16%), Si (2.07%), Mg (4.10%), Fe (3.96%), Sr (2.17%), and Zn (32%), achieved through CF-LIBS were compared with those determined from EDX analysis, showing a good agreement. For the classification of the samples, principal component analysis (PCA) was performed using LIBS spectral data, such as the optical emission lines of Zn (I) at wavelength 481.05 nm, Ca (II) at wavelength 393.37 nm, and C (I) at wavelength 247.86 nm. The first three principal components (PCs) explained 91% of the total variation, including PC1 (58.71%), PC2 (20.9%), and PC3 (11.4%). The present analysis indicates that LIBS with EDX and PCA is an effective and reliable tool for the elemental analysis and classification of plant-biochar samples.

## Figures and Tables

**Table 1:** Major emission lines detected in the emission spectrum of the plant-biochar sample (S1).

*Elements*	*Wavelength λ (nm)*
*Hydrogen (H)*	*656.28 (H_α_)*
*Lithium (Li I)*	*610.35, 670.77, 670.79*
*Carbon (C I)*	*247.86*
*Sodium (Na I)*	*589.59, 568.82, 588.99*
*Magnesium (Mg I)*	*518.36, 517.27, 516.73, 383.83, 383.23, 277.98, 277.67, 278.30, 285.21*
*Magnesium (Mg II)*	*279.80, 280.27, 279.08, 279.55*
*Aluminum (Al I)*	*309.27, 394.40, 308.21, 396.15*
*Silicon (Si I)*	*250.69, 251.43, 251.61, 251.92, 252.41, 252.85, 288.16*
*Calcium (Ca I)*	393.37, 396.85, 422.67, 428.30, 428.94, 429.90, 430.25, 430.77, 431.87, 442.54, 443.50, 445.48, 487.81 *, 527.03 *, 534.95, 558.20, 558.88, 559.01, 559.45, 559.85 *, 585.75 *, 612.22 *, 616.13, 616.22, 643.91, 646.26, 649.97 *, 671.76 *
*Calcium (Ca II)*	*317.93, 373.69, 393.37, 396.85, 315.89, 370.60*
*Iron (Fe I)*	259.94, 300.09, 302.06, 302.58
*Iron (Fe II)*	*256.25, 258.59,* 260.65, 261.18, 261.35, 261.76, 262.54, 262.82, 273.95, 274.32, 274.64, 274.93, 275.57, *294.77*
*Strontium (Sr II)*	*421.55, 407.77*
*Zinc (Zn I)*	280.12 *, 307.59, 328.23, 330.29 *, 334.53, 468.01 *, 472.22 *, 481.05 *, 636.23
*Zinc (Zn II)*	255.79

* Emission spectral lines of calcium and zinc used to construct the typical Boltzmann plots [32].

**Table 2:** Atomic parameters of neutral calcium (Ca I) spectral lines utilized to structure the Boltzmann plot.

*Wavelength λ (nm)*	*Transition* *Upper to Lower*	*Upper-Level Energy* *E_k_ (cm^−1^)*	*Transition Probability and Statistical Weight A_k_g_k_ (10^8^s^−1^)*
*Ca I*			
*487.81*	*4s4f ^1^F_3_ → 3d4s ^1^D_2_*	*42,344.08*	*1.32*
*527.03*	*3d4p ^3^P_2_ → 3d4s ^3^D_3_*	*39,359.83*	*2.5*
*559.85*	*3d4p ^3^D_1_ → 3d4s ^3^D_1_*	*38,230.65*	*1.29*
*585.75*	*4p^2 1^D_2_ → 4s4p ^1^P_1_*	*40,730.97*	*3.3*
*612.22*	*4s5s ^3^S_1_ → 4s4p ^3^P_1_*	*31,536.26*	*0.86*
*649.97*	*4p ^3^F_2_ → 4s ^3^D_2_*	*35,730.34*	*0.41*
*671.76*	*4s5p ^1^P_1_ → 3d4s ^1^D_2_*	*36,698.2*	*0.36*
*Zn I*			
*280.12*	*5d ^3^D_1_ → 4p ^3^P_2_*	*68,579.14*	*0.75*
*330.29*	*4d ^3^D_1_ → 4p ^3^P_1_*	*62,772*	*6*
*468.01*	*5s ^3^S_1_ → 4p ^3^P_0_*	*53,672.24*	*0.48*
*472.22*	*5s ^3^S_1_ → 4p ^3^P_1_*	*53,672.23*	*1.37*
*481.05*	*5s ^3^S_1_ → 4p ^3^P_2_*	*53,672.24*	*2.1*
